# The Hospital Pay of Shorthand-Typists

**Published:** 1912-02-03

**Authors:** 


*To the Editor of* THE HOSPITAL.

### THE HOSPITAL PAY OF SHORTHAND-TYPISTS.

*To the Editor of* THE HOSPITAL.

SIR,—I asked my Board recently to advance the salary of our senior woman shorthand-typist. She started work in the office of a hospital (where the annual expenditure is nearly £40,000) six years ago, at a salary of £50 a year. The salary has advanced by £5 a year up to £80, and now it is to advance by two further sums of £5 a year to a *maximum* of £90 a year.

I shall be very grateful if some of your readers will inform me whether they consider £90 a year an adequate salary for an educated woman, who is an expert shorthand-typist? The hours are from 9.30 to 5 P.M. daily, except Saturday, when they are from nine to one, noon. I am, your obedient servant, HOSPITAL SECRETARY.

